# 

**Published:** 1854-01

**Authors:** 


### CONTENTS.

### ORIGINAL COMMUNICATIONS.

Answer to Dr. Drake's Interrogatories........................................ 81
Proceedings of the American Society of Dental Surgeons,............................ 89
Springing of Plates. By Dr. W. S. Crane,.......................................... 93
Dental Exhibitions. By a Dental Surgeon,.......................................... 95
Extraction of Teeth. By Dr. J. Taylor..............................................102
A Maxim for the Dentist. By Dr. John Harris........................................111
Crystal Gold for Filling Teeth. By J. Taft.........................................113

### SELECTED ARTICLES.

New York Exhibition of the Industry of all Nations.................................120
Application of Artificial Teeth, by the Auroplastic Principle. By Edward Truman.124
Non-Fatal Accidents from Anæsthetic Agents, with Observations. By W. H. Mussey, M. D.........130
Dental Abstracts...................................................................136

### CORRESPONDENCE.

Letter from the Professor of Mechanical Dentistry, of the Ohio Col. of Den. Sur...148
Letter from A. W. French...........................................................149
Letter from K......................................................................150

### EDITORIAL.

Crystal Gold for Filling Teeth.....................................................151
The American Society of Dental Surgeons............................................152
New York Society of Dental Surgeons................................................153
A Remedy for the Warping of Plates.................................................153
New Dental Depot...................................................................154
A New Work for the Dental Profession...............................................154
The Mississippi Valley Association of Dental Surgeons..............................155
Ohio Dental College Association....................................................155
Payments for the Register..........................................................155
Obituary...........................................................................155
Forceps for Extraction of Teeth....................................................155
Drs. Taft & Watt...................................................................156

